# T1 mapping for diagnosis of early cardiac cellular allograft rejection

**DOI:** 10.1016/j.jhlto.2026.100678

**Published:** 2026-09-02

**Authors:** L.P.M.A. Soares, F.G. Marcondes-Braga, R.N. Dantas, B.B. Lima, V.S. Faria, A.A. Scussel, R.C.T. Dantas, S. Mangini, I.W. Campos, L.F.B.C. Seguro, M.S. Avila, F.A. Gaiotto, C.H. Nomura, F. Bacal

**Affiliations:** a Department of Heart Transplant, Heart Institute (InCor), Hospital das Clínicas, University of São Paulo School of Medicine, São Paulo, Brazil; b Hospital Beneficência Portuguesa de São Paulo, São Paulo, Brazil; c Hospital Israelita Albert Einstein, São Paulo, Brazil; d Department of Radiology, Cardiothoracic Imaging Section, Vanderbilt University Medical Center, Nashville, TN

**Keywords:** Heart transplantation, Acute cellular rejection, Cardiac magnetic resonance, T1 mapping, Early post-transplant period

## Abstract

**Background:**

Non-invasive surveillance of acute cellular rejection (ACR) has become a primary focus in heart transplantation (HTx), aiming to reduce the frequency of invasive endomyocardial biopsies (EMB). While cardiac magnetic resonance (CMR) with T1 mapping has shown promise as a diagnostic tool, its application during the early post-transplant phase remains a significant knowledge gap, as perioperative tissue injury can confound imaging interpretation.

**Objective:**

To evaluate the accuracy of cardiac T1 mapping for ACR in heart transplant recipients within the first 60 days post-transplantation, using EMB as the reference standard.

**Methods:**

This prospective single-center study included adult HTx recipients within 60 days post-transplant. All patients underwent CMR with native T1 mapping and extracellular volume (ECV) quantification. EMB served as the gold standard, classifying patients into non-significant (<2R) and significant (≥2R) rejection groups.

**Results:**

Forty-five recipients were included; 14 (31.1%) presented with cellular rejection ≥2R. Baseline characteristics such as recipient mean age (50.1 vs 49.6 years, *p* = 0.79) and the interval from HTx to CMR (32 vs 31.4 days, *p* = 0.86) were comparable between groups. Native T1 (1048 ± 46.2 vs 1006.6 ± 60.6 ms, *p* = 0.028) and ECV (36.8 ± 5.3% vs 33.2 ± 5.5%, *p* = 0.043) were significantly elevated in the ≥2R group. Native T1 demonstrated an AUC of 0.700 for diagnosing rejection ≥2R (cutoff: 1036.5 ms; sensitivity 64.3%, specificity 74.2%, PPV 52.9%, NPV 82.1%), whereas ECV yielded an AUC of 0.726 (cutoff: 37.9%; sensitivity 57.1%, specificity 90.3%, PPV 72.7%, NPV 82.4%). Multivariate analysis identified indexed RV end-diastolic volume and the presence of late gadolinium enhancement (LGE) as independent predictors of rejection. However, a multiparametric approach combining native T1 <1037 ms, ECV <38%, and absence of LGE yielded a NPV of 100% within this small subgroup.

**Conclusion:**

The diagnostic accuracy of standalone T1 mapping for identifying ACR was modest in this early post-HTx period. However, native T1 and ECV were significantly higher in the rejection group, and their integration into a multiparametric approach, including LGE, demonstrated a high NPV in this cohort. These findings highlight CMR’s potential for monitoring myocardial changes even in the early post-transplant stage.

## Background

Acute cellular rejection (ACR) remains a primary challenge to the long-term survival of heart transplant (HTx) recipients. Although endomyocardial biopsy (EMB) continues to be the clinical reference for diagnosis, non-invasive surveillance strategies have emerged as promising alternatives.^1^ Growing evidence supporting donor-derived cell-free DNA (dd-cfDNA), gene expression profiling (GEP), and cardiac magnetic resonance (CMR) suggests these modalities are likely to redefine future guidelines, potentially reducing the necessity for routine EMB.^2^, ^3^, ^4^

However, the initial months following HTx represent a period of particular concern and diagnostic challenge. During this early phase, the transplanted myocardium is subjected to various insults, including donor brain death processes and ischemia-reperfusion injury. The resulting myocardial injury makes diagnosis more challenging, and the limitations of currently available non-invasive methods are more evident within this time window.^5^

The use of GEP is generally recommended 55 days after HTx, which limits its applicability during the period of highest rejection incidence.^6^ In contrast, dd-cfDNA has shown interesting potential, with a high negative predictive value even in the first month post-transplant^3^, ^7^; however, its high cost and limited widespread access still restrict its broader utilization. Current practice tends to initiate monitoring protocols with dd-cfDNA in conjunction with GEP from 8 weeks post-transplant as in the ongoing Surveillance HeartCare Outcomes Registry (SHORE) [NCT03695601].

CMR with T1 mapping has demonstrated promising results for rejection detection.^8^, ^9^, ^10^ In a recent meta-analysis that included eight of the main studies utilizing the method, native T1 showed a sensitivity of 0.86 (95% CI: 0.78-0.92; *p* = 0.011) and a specificity of 0.79 (95% CI: 0.73-0.85; *p* < 0.001), whereas extracellular volume (ECV) demonstrated a sensitivity of 0.90 (95% CI: 0.79-0.97; *p* = 0.088) and a specificity of 0.67 (95% CI: 0.58-0.75; *p* < 0.001).^11^ In addition, a combined T1 and T2 threshold (1029 and 60 ms, respectively) yielded an AUC of 0.946, 100% sensitivity, 85% specificity, and 100% negative predictive value, highlighting its potential as a non-invasive surveillance strategy.^11^

Despite these promising findings, it is well-known that native T1 and ECV values are higher in the early post-transplant phase.^12^ However, there is uncertainty regarding the general applicability of this method and the precise time point at which it becomes clinically reliable. Thus, we conducted a study to evaluate the accuracy and feasibility of T1 mapping on CMR for the diagnosis of acute graft rejection in adult heart transplant patients within the first 60 days after transplantation.

## Methods

This was a single-center, observational, prospective, and cross-sectional study conducted at the Heart Institute of the University of São Paulo, Brazil. Adult HTx recipients were screened within their first 60 days post-surgery and past their first biopsy, from March 1, 2020, to December 31, 2023. Patients were excluded if they presented standard contraindications to CMR, such as claustrophobia, inability to lie supine, or metal devices. Additional exclusion criteria included a creatinine clearance <30 ml/min and any prior history of rejection. This study was approved by the Institutional Ethics Committee (Approval Number: 86764218.1.0000.0068) and all participating patients provided written informed consent.

### Cardiac biopsies

EMBs were performed according to institutional protocol (at 15 days and 1, 3, 6, 9, and 12 months post-HTx). For this study, we included only 1 biopsy per patient within the first 60 days post-transplantation. The 15-day biopsy was excluded to mitigate the confounding effects of early post-surgical inflammation and imaging artifacts. This target screening window typically coincided with the patient's second routine surveillance biopsy (scheduled around 30 days). All samples were processed with hematoxylin and eosin staining, alongside immunohistochemical evaluation for C4d and CD68, and were interpreted as part of the institutional routine. Histopathological reports were provided by an anatomical pathologist blinded to the CMR results.

Rejection was graded as 0R, 1R, 2R, or 3R in accordance with the International Society for Heart and Lung Transplantation (ISHLT) criteria.^13^ Based on the EMB results, patients were allocated into 2 groups: nonsignificant rejection (< 2R) and significant rejection (≥ 2R). In patients with a prior history of Chagas disease who presented with significant rejection, additional PCR testing and immunohistochemistry were performed to evaluate for disease reactivation.

### Image acquisition and interpretation

CMR examinations were planned to be performed within 24 hours of the surveillance EMB. In patients treated for ACR, paired CMR and EMB assessments were repeated after treatment whenever feasible. To ensure standardization of parametric indices, a control group of healthy volunteers, matched for age, sex, and race, was recruited to establish local normative values for native T1 and ECV mapping. As these values are highly dependent on scanner environments and post-processing algorithms, this cohort served solely as a technical reference standard. They were not included in the comparative analyses of the transplant recipients.

The images were acquired using a 1.5 T scanner (Titan, Canon Medical System Corporation, Otawara, Japan). Cine imaging and late gadolinium enhancement (LGE) imaging were performed according to standard protocols. T1 mapping was conducted using the Modified Look-Locker Inversion Recovery sequence with a 3(3)3(3)5 sampling scheme. Key imaging parameters included: slice thickness of 10 mm, field of view of 300×300 mm, matrix of 152×150, flip angle of 40°, minimum inversion time (TI) of 60 ms, and a TI increment of 150 ms. Native T1 maps were initially obtained from 3 short-axis views (basal, mid-ventricular, and apical segments) before contrast administration. Post-contrast T1 maps were acquired 15 min after an intravenous bolus injection of 0.2 mmol/kg of gadolinium-based contrast agent (Dotarem, Guerbet Aulnay-Sous-Bois, France).

All CMR images were analyzed using dedicated software (cvi42, Circle Cardiovascular Imaging Inc., Calgary, Canada) by a trained radiologist with >10 years of cardiovascular imaging experience, blinded to EMB results. End-systolic, end-diastolic volumes, LV mass, and biventricular ejection fraction were measured by standard methods.^14^ For qualitative and quantitative assessment of myocardial edema, a T2-weighted triple IR sequence was used, measuring LV myocardium signal in the SA and adjacent skeletal muscle. The T2 signal intensity (SI) ratio was calculated as T2 SI = myocardiumSI / muscleSI. Myocardial edema was diagnosed when the myocardial T2 SI ratio was >1.9.^15^, ^16^

LGE images were qualitatively and quantitatively analyzed for its presence and fibrosis mass, its pattern (e.g., subendocardial, mid-wall, subepicardial, transmural) and regional distribution according to the 17-segment AHA model, excluding the apex. For T1 mapping and ECV estimation, mid-septal regions of interest were carefully drawn on short-axis images to avoid contamination from the left ventricular outflow tract and contrast in thinner myocardial layers. ECV was calculated using pre-contrast and post-contrast myocardial and blood T1 values as recommended.^14^

### Statistical analysis

The sample size was calculated based on the diagnostic accuracy of native T1 for acute rejection, using the area under the receiver operating characteristic curve (AUC) as the primary measure. Based on a reference study^8^ reporting an AUC of 0.89 and a rejection prevalence of 25% and considering 80% power and a 2-sided significance level of 0.05, a minimum of 40 patients was determined necessary for inclusion.

All statistical analyses were performed using R software version 4.2.2 (R Core Team, 2022) with the pROC package. Quantitative variables were presented as mean ± standard deviation or median [Q1; Q3] based on normality assessed by the Kolmogorov-Smirnov test. Categorical variables were expressed as frequencies and percentages. For group comparisons, the independent *t*-test (or ANOVA) was used for normally distributed quantitative data, while the Wilcoxon-Mann-Whitney test was used for non-normally distributed data. For categorical variables, comparisons were made using Fisher's exact test or the chi-square test. A 2-sided significance level of 5% (α=0.05) was adopted for all statistical tests.

Univariate logistic regression was performed to assess the association of each variable with acute cellular rejection. Subsequently, multiple logistic regression was conducted using a stepwise backward selection method, with entry and exit criteria set at 5%. Variables with a *p*-value < 0.1 in the univariate analysis were included in the model. Due to the high correlation between right ventricular end-diastolic and end-systolic volumes, only the diastolic was retained for the multivariate analysis.

The diagnostic accuracy of native T1 and ECV measurements was assessed by generating ROC curves and determining their respective AUCs, sensitivities, and specificities with 95% confidence intervals.

## Results

**Figure 1:**
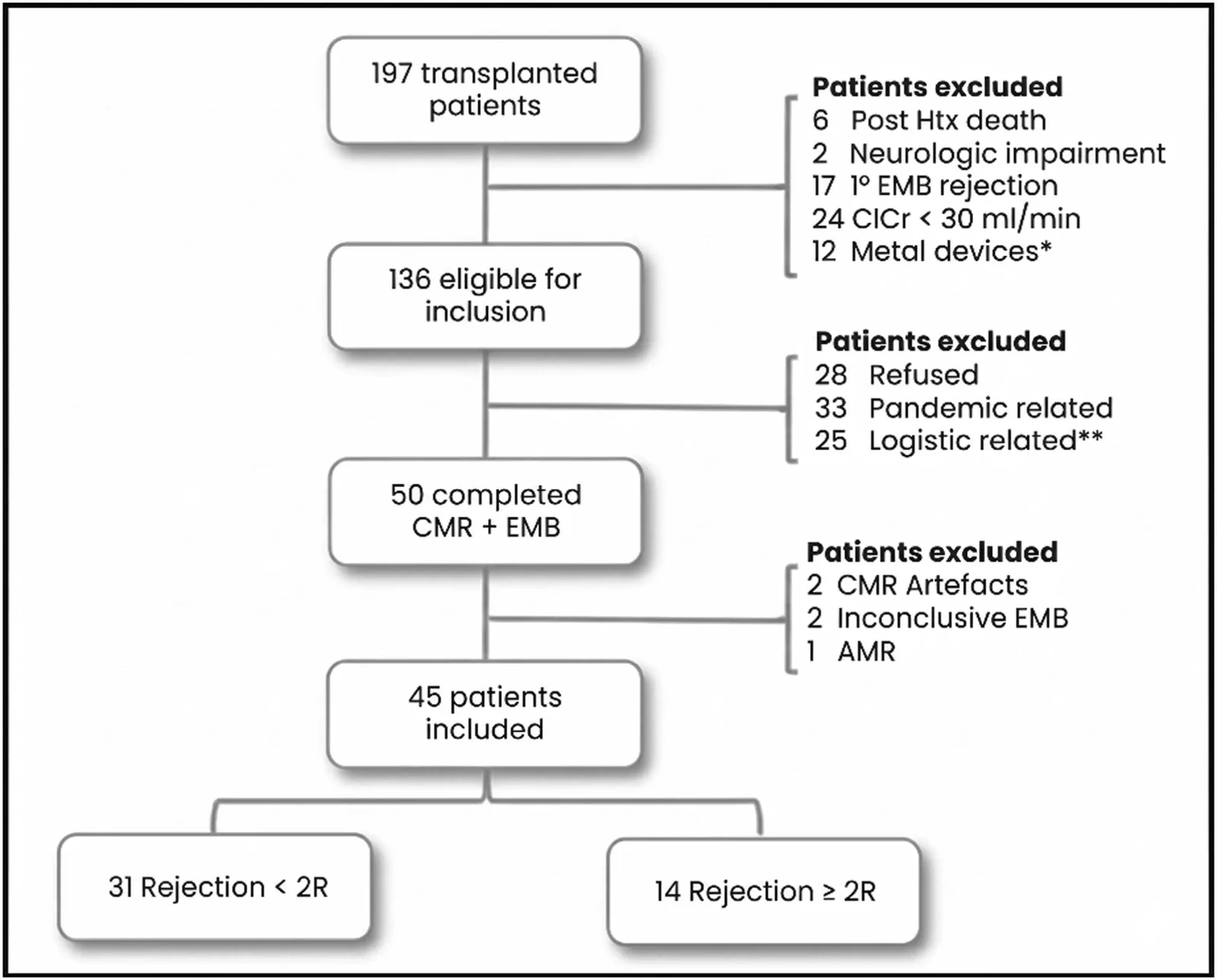
Study flow diagram. HTx, heart transplant; EMB, endomyocardial biopsy; ClCr, creatinine clearance; CMR, cardiac magnetic resonance; AMR, antibody mediated rejection. * 5 retained pacemaker leads, 3 unidentified surgical clips, 3 extensive thoracic tattoos, and 1 retained projectile fragment. ** Refers to equipment downtime, scheduling constraints within the study protocol window, and operational limitations inherent to public health service demand.

A total of 197 adult patients underwent HTx during the study period. After applying all exclusion criteria and overcoming inclusion challenges, 45 recipients constituted the final study cohort, as detailed in Figure 1. It is important to note that during the study period, patients with COVID-19 or those who were contacts of confirmed cases were excluded, and patient screening was intermittently interrupted due to pandemic-related restrictions. Furthermore, overcoming the inclusion challenges inherent to a high-volume public health center in a developing nation represented a significant hurdle. A total of 25 patients were excluded due to logistical constraints such as: high institutional demand causing scheduling conflicts (13 patients) and periods of downtime of the dedicated research scanner (12 patients). Only 1 patient had AMR, which was classified as pAMR1 (I+) concurrent with mild acute cellular rejection (ACR 1R). Given this very low prevalence, we chose to exclude this patient to maintain a homogeneous cohort focused strictly on ACR.

EMB results demonstrated the following distribution of ACR grades: 12 patients (27.7%) were classified as 0R, 19 (42.2%) as 1R, and 14 (31.1%) as 2R. Notably, no cases of ACR grade 3R or Chagas disease reactivation were identified within the cohort. The baseline demographic and clinical characteristics for the resultant non-significant (<2R) and significant (≥2R) rejection groups are summarized in Table 1. Notably, the mean interval from HTx to CMR was 31.8 ± 9.8 days, with no difference between groups, highlighting the high-risk early postoperative window during which these examinations were performed.

**Table 1:** Baseline Characteristics of Significant and Non-significant Rejection Groups

	Rejection < 2R (*n* = 31)	Rejection ≥ 2R (*n* = 14)	Total (*n* = 45)	*p* value
Male (*n*, %)	18 (58.1)	8 (57.1)	26 (57.8)	>0.999
Recipient age (years)	49.4 ± 10.1	50.1 ± 8.5	49.6 ± 9.6	0.797
Etiology (*n*, %)				0.836
Chagasic	8 (25.8)	5 (35.7)	13 (28.9)	
Ischemic	5 (16.1)	1 (7.1)	6 (13.3)	
Dilated	15 (48.4)	6 (42.9)	21 (46.7)	
Hypertrophic	1 (3.2)	1 (7.1)	2 (4.4)	
Hypertensive	1 (3.2)	0 (0.0)	1 (2.2)	
Peripartum	1 (3.2)	1 (7.1)	2 (4.4)	
ClCr (ml/min/1.73m^2^)	72.4 ± 24.9	68.1 ± 22.9	71.1 ± 24.1	0.570
PRA > 10% (*n*, %)	3 (10)	2 (13.3)	5 (11.1)	>0.999
Recipient ABO type (*n*,%)				0.859
A	12 (38.7)	5 (35.7)	17 (37.8)	
B	6 (19.4)	2 (14.3)	8 (17.8)	
O	13 (41.9)	7 (50.0)	20 (44.4)	
Donor age	28.8 ± 9.6	31.2 ± 8.7	29.6 ± 9.3	0.413
Ischemia time (hours)	3:08 ± 1:13 (*n* = 28)	3:09 ±1:20 (*n* = 11)	3:08 ± 1:14 (*n* = 39)	0.851
PGD (*n*, %)	2 (6.5)	3 (21.4)	5 (11.1)	0.166
Interval HTx/CMR (days)	32.0 ± 9.9	31.4 ± 9.9	31.8 ± 9.8	0.867
Interval CMR/EMB (days)	−0.6 ± 3.0	0.0 ± 4.8	−0.4 ± 3.6	0.663
Per cause Biopsy (*n*,%)	2 (6.5)	1 (7.1)	3 (6.7)	>0.999
ISS (*n*, %)				
Corticosteroid	31 (100.0)	14 (100.0)	45 (100.0)	>0.999
Cyclosporine	29 (93.5)	13 (92.9)	42 (93.3)	>0.999
Tacrolimus	2 (6.5)	1 (7.1)	3 (6.7)	>0.999
Mycophenolate	23 (74.2)	9 (64.3)	32 (71.1)	0.502
Azathioprine	8 (25.8)	5 (35.7)	13 (28.9)	0.502

ClCr, Creatinine clearance; PRA, Panel reactive antibodies; PGD, Primary graft dysfunction; CMR, Cardiac magnetic resonance; EMB, Endomyocardial biopsy; ISS, immunosuppressive agent.

When comparing CMR functional findings between the groups, a statistically significant difference was observed in right ventricular variables. The right ventricular ejection fraction (RVEF) was lower in the group with significant rejection (≥ 2R) (53.1% ± 7.4 vs 58.6% ± 7.3; *p* = 0.029), and both the indexed right ventricular end-diastolic volume (RVEDVi) and the indexed right ventricular end-systolic volume (RVESVi) were significantly larger in this group (Table 2).

**Table 2:** Cardiac Magnetic Resonance Functional and LGE Findings of Significant and Non-significant Rejection Groups

	Rejection < 2R (*n* = 31)	Rejection ≥ 2R (*n* = 14)	*p*-value
LVEF (%)	63.4 ± 6.3	62.4 ± 9.7	0.721
LVEDVi (ml/m²)	71.0 ± 13.3	80.8 ± 19.8	0.107
LVESVi (ml/m²)	26.1 ± 7.7	31.7 ± 15.8	0.224
RVEF (%)	58.6 ± 7.3	53.1 ± 7.4	**0.029**
RVEDVi (ml/m²)	68.5 ± 21.1	85.7 ± 24.1	**0.031**
RVESVi (ml/m²)	27.5 ± 10.1	40.1 ± 16.0	**0.014**
LAVi (ml/m²)	40.9 ± 16.3	51.7 ± 18.6	0.087
LGE	13 (41.9%)	12 (85.7%)	**0.009**
N° segments	5.7 ± 3.3 (*n* = 13)	3.0 ± 2.7 (*n* = 12)	0.035
Mass (g)	13.1 ± 13.3 (*n* = 13)	6.5 ± 9.9 (*n* = 12)	0.170
T2 edema	11/31 (35.5%)	9/13 (69.2%)	0.053
N° segments	4.5 ± 2.3 (*n* = 11)	3.0 ± 2.7 (*n* = 9)	0.218
T2 ratio > 1.9	10/11 (90.9%)	8/9 (88.9%)	>0.999

LVEF, Left Ventricular Ejection Fraction; LVEDVi, Left Ventricular End-Diastolic Volume index; LVESVi, Left Ventricular End-Systolic Volume index; RVEF, Right Ventricular Ejection Fraction; RVEDVi, Right Ventricular End-Diastolic Volume index; RVESVi, Right Ventricular End-Systolic Volume index; LAVi, Left Atrial Volume index; LGE, Late gadolinium enhancement. Bold value indicates statistical significance (p < 0.05).

As for the presence of LGE, it was significantly higher in the rejection ≥ 2R group (85.7% vs 41.9%; *p* = 0.009). Only 1 patient with non-significant rejection showed subendocardial and papillary LGE, while all the others in both groups exhibited a non-coronary enhancement pattern. Despite the increased occurrence of LGE in the ≥2R group, the number of affected myocardial segments was significantly higher in the patients from the <2R group who did present LGE (*p* = 0.035). Additionally, T2-weighted imaging showed a trend toward a higher prevalence of edema in the ≥2R group (Table 2).

**Figure 2:**
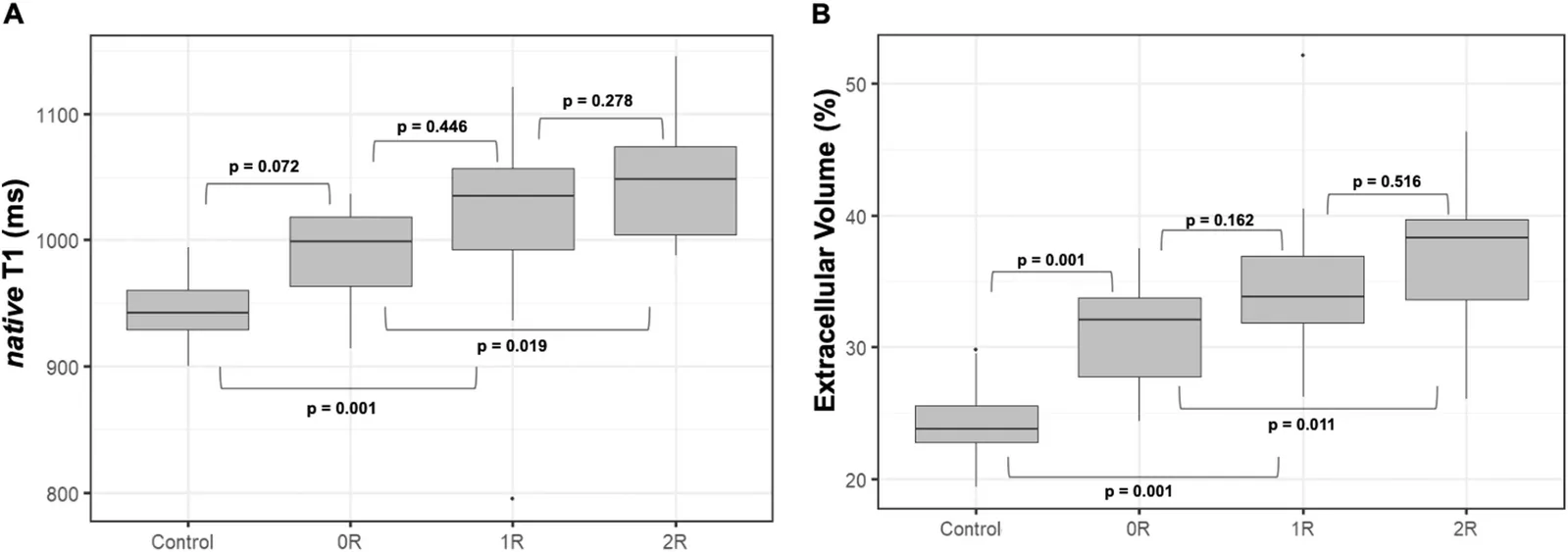
Comparison of T1 mapping values across the Control, 0R, 1R, and 2R groups. **(A)** Boxplots of native T1 values (ms) for each group, showing a progressive increase with rejection grade. **(B)** Boxplots of Extracellular Volume (ECV) (%) for each group, also demonstrating an increase proportional to rejection severity. An initial ANOVA revealed a significant difference between groups (*p* < 0.001), with specific pairwise differences shown by the *post-hoc* Tukey test in the connecting lines.

The mean native T1 values were 945.9 ± 25.0 ms for the control group, 990.1 ± 36.3 ms for 0R, 1016.9 ± 70.9 ms for 1R, and 1048.0 ± 46.2 ms for 2R. As for the ECV, the mean values were 24.4% ± 2.7% for the control group, 31.0% ± 4.1% for 0R, 34.5% ± 5.8% for 1R, and 36.8% ± 5.3% for 2R. A consistent trend was observed where native T1 and ECV values increased with the severity of rejection. The 2-by-2 comparisons among these groups further illustrate these differences and are presented in detail in Figure 2.

**Figure 3:**
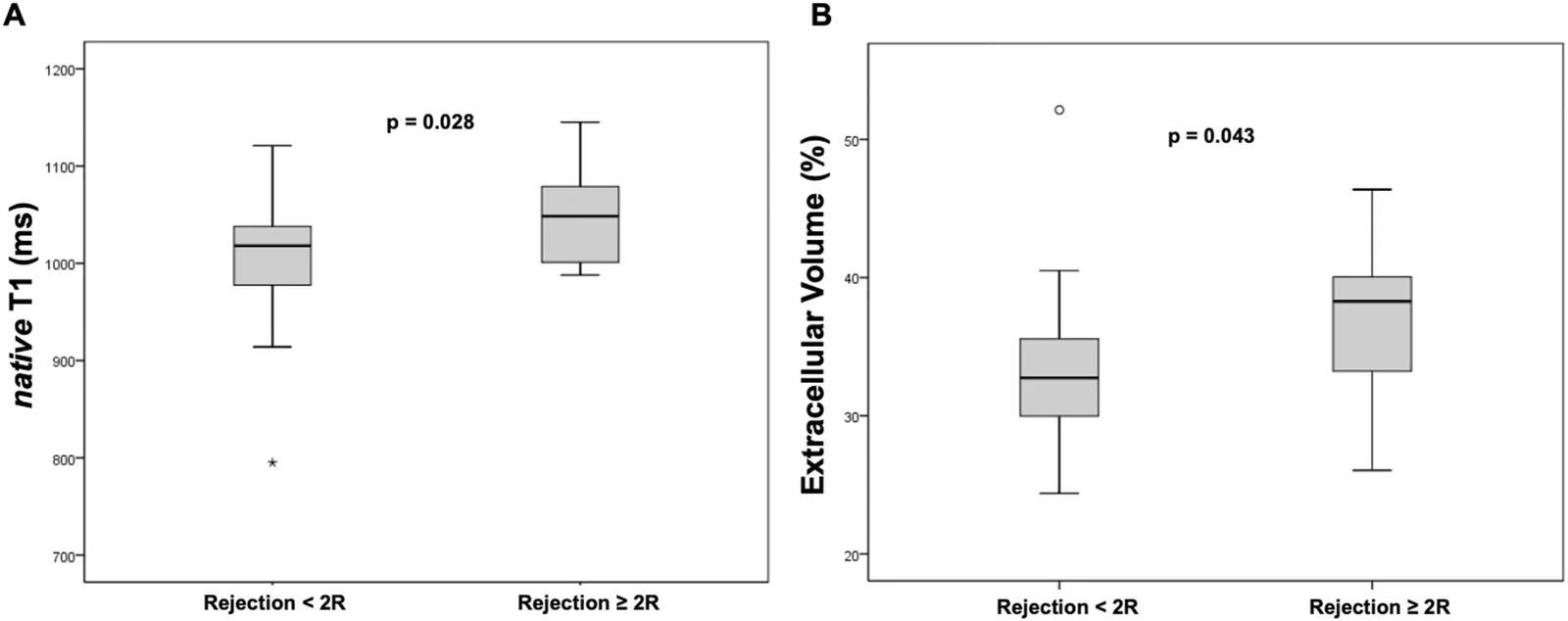
Comparison of native T1 and ECV values between rejection groups. Box plots showing: **(A)** native T1 values in milliseconds (ms), and **(B)** Extracellular Volume in percentage (%) for the <2R and ≥2R rejection groups.

A comparison between the ≥2R and <2R rejection groups revealed that native T1 and ECV values were significantly higher in the former group (native T1: 1048 ± 46.2 ms vs 1006.6 ± 60.6 ms, *p* = 0.028; ECV: 36.8% ± 5.3% vs 33.2%±5.5%, *p* = 0.043) (Figure 3).

**Figure 4:**
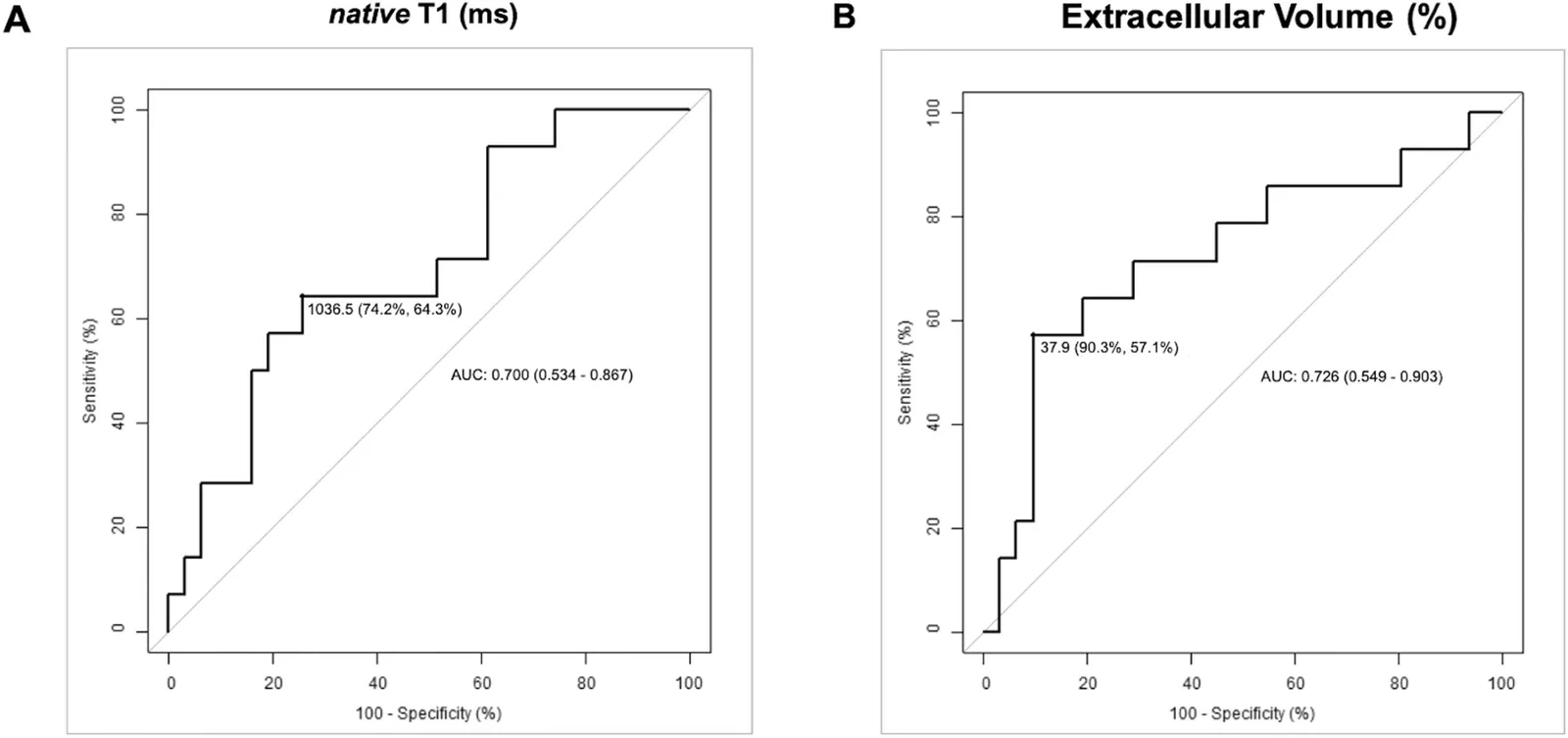
Diagnostic accuracy analysis of T1 mapping for significant rejection. **(A)** Receiver operating characteristic (ROC) curve for native T1. **(B)** ROC curve for extracellular volume.

To assess diagnostic accuracy, ROC curves for native T1 and ECV were generated, and their respective area under the curve (AUC) values were determined. For native T1, the AUC was 0.700 (95% confidence interval: 0.534-0.867). Using a cutoff point of 1036.5 ms, the sensitivity was 64.3%, specificity was 74.2%, positive predictive value was 52.9%, and negative predictive value was 82.1%. For ECV, the AUC was 0.726 (95% confidence interval: 0.549-0.903). A cutoff point of 37.9% resulted in a sensitivity of 57.1%, specificity of 90.3%, positive predictive value of 72.7%, and negative predictive value of 82.4% (Figure 4).

**Figure 5:**
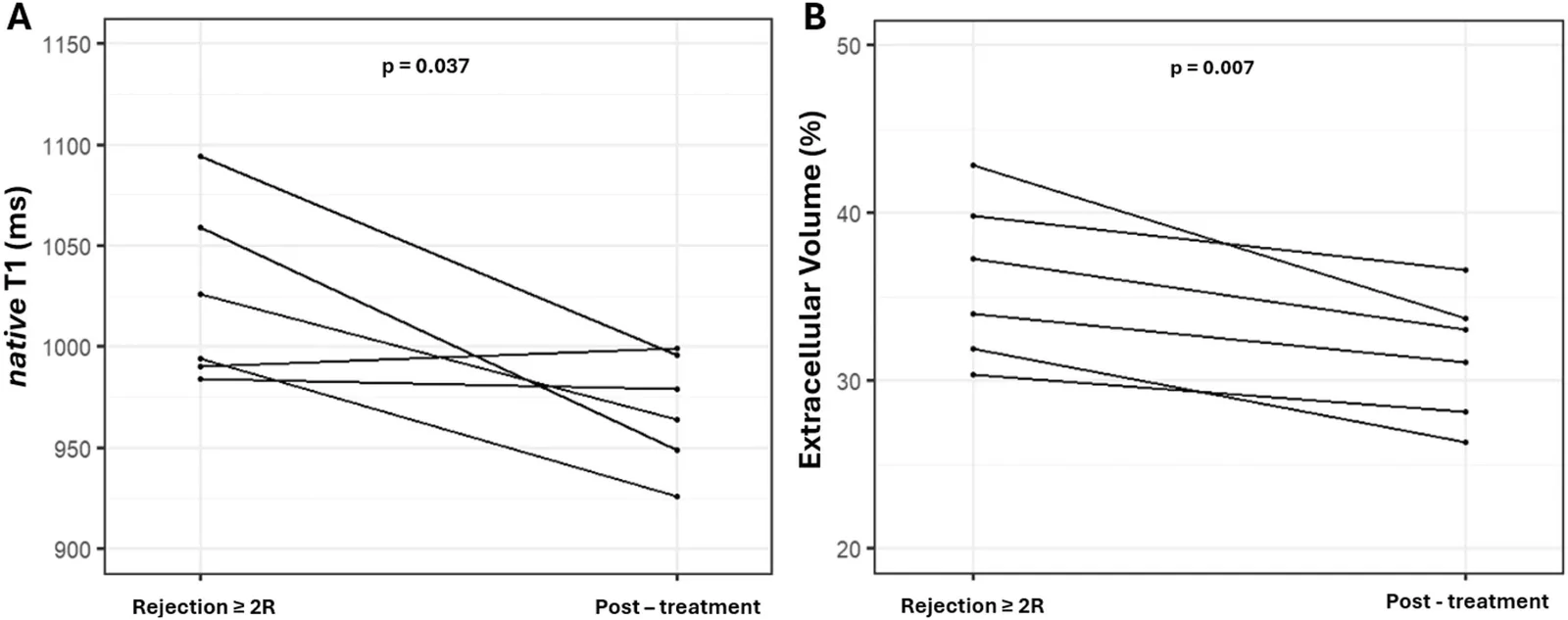
Paired comparison of **(A)** native T1 values (ms) and **(B)** extracellular volume (ECV) fraction (%) in the longitudinal subgroup (*n* = 6) pre- and post-treatment of significant acute cellular rejection episode (≥2R).

Among the 14 patients with ACR ≥ 2R, 6 patients underwent a repeat CMR examination following treatment. Longitudinal follow-up was missed in the remaining 8 patients due to scheduling conflicts or protocol refusal. Figure 5 illustrates the paired pre- and post-treatment measurements. A statistically significant reduction was observed after treatment for both native T1 values (1024.5 ± 44.2 vs 968.8 ± 28.3; *p* = 0.037) and ECV (36.0 ± 4.8 vs 31.5 ± 3.8; *p* = 0.007).

A logistic regression analysis, adjusted for pre-selected variables, was performed to identify independent predictors of rejection. In the univariate analysis, several variables demonstrated a statistically significant association with rejection, including right ventricular ejection fraction, indexed right ventricular end-diastolic volume, LGE, T2 edema, and native T1. In the final multivariate analysis, the 2 variables that remained as independent predictors of rejection were RVEDVi and LGE, with the presence of LGE demonstrating the highest odds ratio (Table 3).

**Table 3:** Logistic Regression Analysis for Independent Predictors of Rejection.

	Univariate			Multivariate		
	Odds ratio	95% CI	*p*-value	Odds ratio	95% CI	*p*-value
RVEF (%)	0.890	0.800-0.990	**0.032**			
RVEDVi (ml/m^2^)	1.034	1.003-1.066	**0.029**	1.036	1.001-1.073	**0.048**
LAVi (ml/m^2^)	1.036	0.996-1.077	0.076			
LGE	8.31	1.58-43.61	**0.012**	5.987	1.054-34.024	**0.044**
LGE mass (g)	1.001	0.940-1.065	0.980			
T2 edema	4.09	1.02-16.40	**0.047**			
Native T1 (ms)	1.017	1.001-1.032	**0.034**			
ECV (%)	1.134	0.996-1.291	0.058			

RVEF, Right ventricular ejection fraction; RVEDVi, Indexed right ventricular end-diastolic volume; LAVi, Indexed left atrial volume; LGE, Late Gadolinium Enhancement; ECV, Extracellular Volume. Bold value indicates statistical significance (p < 0.05).

**Figure 6:**
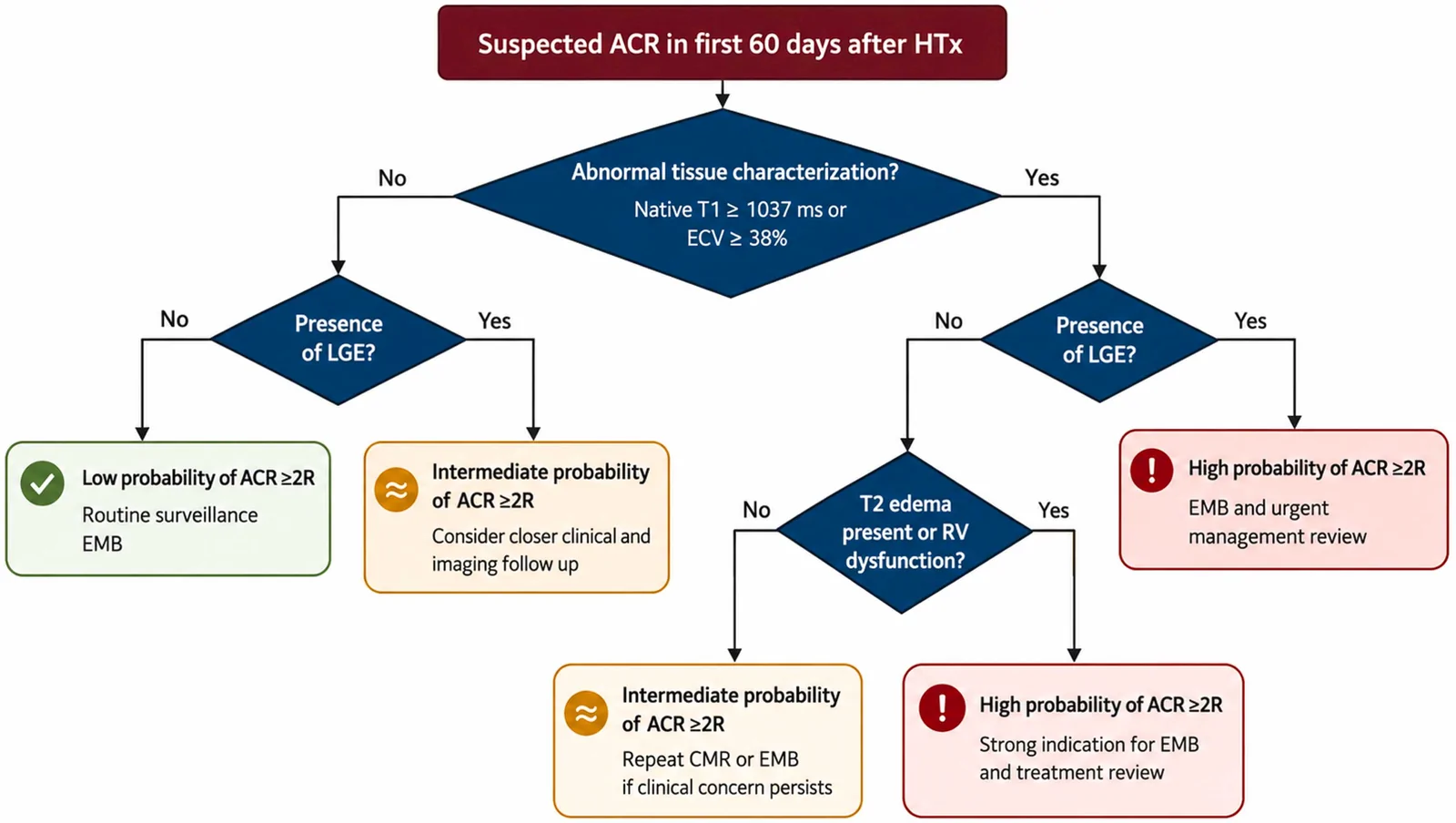
*Proposed multiparametric CMR algorithm for ACR surveillance in the first 60 days post-HTx.* ACR, acute cellular rejection; CMR, cardiac magnetic resonance; ECV, extracellular volume; EMB, endomyocardial biopsy; HTx, heart transplantation; LGE, late gadolinium enhancement; NPV, negative predictive value; RV, right ventricle.

Based on the interpretation of our data, CMR parameters were integrated into a sequential, multiparametric diagnostic algorithm (Figure 6), with its performance detailed in Table 4. The key clinical strength of this model lies in its negative evaluation pathway (normal tissue characterization and absent LGE). In this subgroup, which comprised one-third of the entire cohort (*n* = 15), the pathway yielded an AUC of 0.841 and an observed NPV of 100% (95% CI: 78.2%-100.0%). Finally, although the combined framework (*n* = 5) yielded perfect sensitivity and specificity, the very small sample size and wide confidence intervals indicate that these exploratory results are not definitive and require cautious interpretation.

**Table 4:** Diagnostic Performance of the Multiparametric CMR Pathways for Detecting Acute Cellular Rejection

Diagnostic Pathway	*n* (%)	Sensitivity	Specificity	PPV	NPV	AUC
*Abnormal Tissue Characterization (Native T1 ≥ 1037 ms or ECV ≥ 38%)*	19 (42.2%)	71.4% (41.9%-91.6%)	71.0% (52.0%-85.8%)	52.6% (28.9%-75.6%)	84.6% (65.1%-95.6%)	0.712 (0.565-0.859)
*Negative Pathway Evaluation (Normal T1/ECV AND Negative LGE)*	15 (33.3%)	100.0% (39.8%-100.0%)	68.2% (45.1%-86.1%)	36.4% (10.9%-69.2%)	100.0% (78.2%-100.0%)	0.841 (0.741-0.941)
*Positive Pathway Evaluation (Abnormal T1/ECV AND Positive LGE)*	14 (31.1%)	80.0% (44.4%-97.5%)	33.3% (7.5%-70.1%)	57.1% (28.9%-82.3%)	60.0% (14.7%-94.7%)	0.567 (0.358-0.776)
*Combined framework (Abnormal T1/ECV + Negative LGE AND T2 edema or RV dysfunction)*	5 (11.1%)	100.0% (15.8%-100.0%)	100.0% (29.2%-100.0%)	100.0% (15.8%-100.0%)	100.0% (29.2%-100.0%)	1.000 (1.000-1.000)

AUC, area under the curve; CMR, cardiac magnetic resonance; ECV, extracellular volume; LGE, late gadolinium enhancement; NPV, negative predictive value; PPV, positive predictive value; RV, right ventricle.

## Discussion

This study evaluated the diagnostic accuracy of T1 mapping (in CMR) for detecting ACR compared to EMB during the early post-HTx phase. While the diagnostic accuracy was modest, significant elevations in both native T1 and ECV were consistently observed in rejection cases.

While there is a growing pursuit of non-invasive methods for the diagnosis of ACR, their applicability remains limited during the high-risk early post-transplant period (first 60 days), when baseline tissue injury often complicates imaging interpretation. In our study cohort, we addressed this critical diagnostic gap, as the mean time from transplantation to CMR was 31.8 ± 9.8 days. Even though prior literature has included some patients in earlier phases, these studies generally lacked a specific focus on this challenging initial window.^2^, ^5^, ^17^ To our knowledge, this is the first study exclusively dedicated to the immediate post-transplant phase.

Remarkably, the accuracy of native T1 and ECV was lower than what has been reported in the literature.^4^, ^8^, ^10^, ^11^ Anthony et al^4^ investigated CMR surveillance starting at 4 weeks post-transplant and demonstrated that a CMR-based strategy was non-inferior to EMB, reducing the need for invasive biopsies by 94%. In their analysis of 401 CMR scans, native T1 achieved an AUC of 0.897 and a negative predictive value (NPV) of 98.7% for detecting ≥2R rejection. However, their study design spanned a much longer period (up to 52 weeks), and the number of high-grade rejection events in the early phase was notably low (e.g., only 1 event per group at 4 weeks). This suggests that the superior accuracy observed in their cohort may be driven by data from later phases, when the confounding effect of early post-transplant myocardial injury is less pronounced. Therefore, while T1 mapping shows promise, its performance appears to be more robust in later stages when post-surgical inflammation has subsided.

Nevertheless, even in this initial phase, we observed a significant difference in T1 mapping values between the groups with and without significant rejection (Figure 3). Furthermore, we demonstrated a clear trend of increasing native T1 and ECV values correlating with the severity of rejection grades (Figure 2). Additionally, despite the small sample size, our longitudinal data showed a significant reduction in both measures following anti-rejection therapy (Figure 5), suggesting a potential utility of this method in monitoring treatment response. These findings reinforce the potential of these parameters as indicators of myocardial injury.

Consistent with our results, Imran et al^8^ reported significantly elevated native T1 values in high-grade rejection (1,052 ± 69 ms), a magnitude very similar to our findings (1,048.0 ± 46.2 ms). However, a key distinction lies in the timing of the assessment: their study recruited patients starting from 6 weeks post-transplantation (with a mean interval of 10 weeks). Our study addresses a critical gap by focusing on the immediate post-transplant phase (mean 31 days), demonstrating that T1 signal alterations are detectable even in this very early window despite the higher background of surgical inflammation.

Additionally, the diagnostic performance of T1 mapping may be enhanced by a multimodal approach. Sade et al^10^ highlighted the value of combining native T1 with echocardiographic Global Longitudinal Strain. They found that a combination of Global Longitudinal Strain > −16% and T1 ≥ 1060 ms identified rejection with 91% sensitivity and a 92% NPV. Collectively, these findings suggest that T1 mapping, especially when integrated into a multiparametric protocol, holds significant promise for non-invasive surveillance, supporting the trend toward reducing unnecessary biopsies.

Regarding functional and volumetric analysis, we observed that RV parameters were significantly associated with early rejection. Specifically, rejection cases demonstrated a lower ejection fraction (*p* = 0.029) and larger indexed end-diastolic and end-systolic volumes (*p* = 0.031 and *p* = 0.014, respectively). Bacal et al^18^ have shown that RV volumes and mass are usually increased in early phases of HTx. However, prior echocardiographic studies in adults and recent CMR data in pediatric cohorts have also identified RV dysfunction as an indicator of rejection.^19^, ^20^, ^21^ These findings suggest that early rejection may disproportionately impact RV function and morphology, positioning it as a relevant marker for allograft dysfunction.

The presence of LGE was significantly more common in patients with rejection ≥ 2R (85.7% vs 41.9%, *p* = 0.009). LGE is a well-established marker of myocardial fibrosis, and in the post-transplant setting, it has been associated with worse prognosis.^22^ However, it does not always indicate irreversible injury and can represent interstitial expansion and tissue edema.^23^ It has been shown that in some cases of myocarditis the LGE can completely resolve in follow-up CMR, showing its value on the diagnosis of acute conditions.^24^ Although some studies have shown an increase in LGE during acute rejection,^5^, ^25^ its overall diagnostic accuracy has been inconsistent. A meta-analysis, for example, found LGE to have a weak sensitivity of 50.1%, a specificity of 60.2%, and an AUC of 0.560. Our study is among the first to evaluate LGE in this very early post-transplant phase. It is known that LGE is more frequent in the earlier phases due to various factors,^26^ and ACR could be an important one.

A surprising finding in our cohort was that while LGE was more frequent in patients with rejection ≥2R, the extent of enhancement was significantly greater in the non-significant rejection group. This observation suggests that the focal nature of LGE, which relies on the presence of relatively healthy myocardium to generate visual contrast, may underestimate more subtle, diffuse inflammatory processes. It is plausible that the more diffuse enhancement observed in patients without significant rejection reflects tissue alterations inherent to the early postoperative period, such as ischemia-reperfusion injury and surgical manipulation, which may manifest diffusely during this time window. Conversely, in the setting of ACR, LGE appears to manifest in a more localized fashion, potentially causing the technique to miss areas of microscopic interstitial injury. In this scenario, T1 mapping, as an absolute quantification technique, plays a relevant complementary role. It does not depend on relative tissue contrast and tends to be more sensitive to the diffuse edema characteristic of rejection. Although our direct analysis did not demonstrate isolated statistical superiority for this purpose, the convergence of these findings with existing literature reinforces the necessity of a multiparametric assessment to overcome the individual limitations of each CMR sequence.

In the multivariate logistic regression analysis, increased RVEDVi and the presence of LGE emerged as the only independent predictors of rejection ≥ 2R. Although native T1 and ECV demonstrated statistically significant differences in univariate analyses, they did not retain significance in the final multivariate model. This phenomenon may be attributed to the interdependence of these variables. While native T1 and ECV reflect interstitial expansion and edema, RVEDVi and LGE may indicate more advanced structural changes and established myocardial injury, potentially accounting for the variance otherwise explained by the parametric mapping variables in the multivariate model. This observation does not invalidate the utility of T1 mapping; rather, it suggests that in the complex early post-transplant phase, markers of evident structural repercussion and macroscopic tissue injury carry preponderant predictive weight. Furthermore, the statistical power to identify multiple independent predictors may have been limited by the total number of rejection events in this cohort, suggesting that tissue mapping may yet play an important role in predictive models derived from larger populations.

The clinical utility of native T1 and ECV may be improved when these measures are integrated into a multiparametric “rule-out” framework (Figure 6). In our cohort, the combination of normal tissue characterization (native T1 < 1037 ms and ECV < 38%) and the absence of LGE yielded a NPV of 100% (Table 4). This high NPV suggests a potential strategy to reduce the reliance on invasive surveillance.

However, extreme caution must be taken when interpreting this diagnostic flowchart, primarily due to our limited sample size and the fact that this high NPV derives from a small subgroup. While the point estimates across several nodes appear highly promising, it is critical to emphasize that these findings possess only internal validity at this stage. This decision-making framework cannot be broadly generalized without extensive external validation in larger, multi-center cohorts. Consequently, these preliminary results are currently insufficient to recommend the omission or postponement of EMB during this early post-transplant phase, and routine EMB remains the standard of care.

Looking ahead, the future of cardiac allograft rejection assessment undoubtedly lies in a multimodal surveillance strategy. While T1 mapping demonstrated modest stand-alone accuracy during this specific early window, it is likely to play a crucial complementary role alongside other non-invasive markers, such as GEP and dd-cfDNA. Furthermore, the clinical utility of quantitative imaging is poised for significant enhancement through the integration of Artificial Intelligence (AI) and machine learning protocols. AI can be leveraged to automatically analyze multiparametric CMR data and identify subtle patterns invisible to standard analysis. This technological evolution has the potential to scale diagnostic capabilities across diverse cohorts, cementing T1 mapping’s position as a robust component of comprehensive clinical surveillance.

## Limitations

This was a single-center study with a relatively small number of patients and CMR examinations, which may limit the generalizability of our results to other transplant centers. The study design, with a limited number of events, may also have reduced the statistical power of the multivariate analysis, and the wide confidence intervals observed in our data further constrain the strength of our conclusions. Additionally, there was inherent variability in the timing between CMR and EMB (mean interval −0.4 ± 3.6 days). Consequently, although the protocol aimed at a 24-hour window, not all examinations were performed strictly within this timeframe. Given that acute cellular rejection can be a dynamic, rapidly evolving process, this interval variation may affect the direct correlation between imaging findings and the histological state at the time of biopsy. Furthermore, a quantitative assessment of T2 mapping was not performed due to technical limitations, which, as shown in other studies, can provide valuable complementary information to T1 mapping for the diagnosis of rejection. Future multicenter studies with larger patient cohorts are warranted to confirm our findings and to explore the incremental value of a multiparametric approach in the early post-transplant period.

## Conclusions

The diagnostic accuracy of standalone T1 mapping for early cellular rejection was modest. However, the significant elevation of native T1 and ECV in the rejection group, alongside a clear trend of increasing values correlating with rejection severity, highlights CMR’s potential for monitoring myocardial changes even in the early post-transplant stage. Furthermore, when integrated into a multiparametric approach, specifically in the absence of LGE, the method demonstrated a high NPV. Although CMR may provide useful complementary information, large-scale prospective studies are essential to establish its definitive role in routine clinical surveillance in this period.

## Disclosure statement

The authors report no conflicts of interest.

## Financial support

Canon Medical Systems of Brazil.

## Declaration of Generative AI and AI-Assisted Technologies in the Writing Process

During the preparation of this work the authors used ChatGPT (OpenAI) in order to improve language and readability, with caution. After using this tool/service, the authors reviewed and edited the content as needed and take full responsibility for the content of the publication.

## Declaration of Competing Interest

The authors declare the following financial interests/personal relationships, which may be considered as potential competing interests: Luis Paulo de Miranda Araujo Soares reports financial support was provided by Canon Medical Systems of Brazil. If there are other authors, they declare that they have no known competing financial interests or personal relationships that could have appeared to influence the work reported in this paper.
